# Providing holistic care for patients with metabolic dysfunction‐associated steatotic liver disease/metabolic dysfunction‐associated steatohepatitis: Key aspects of clinical assessment and how to develop individualised care plans for surveillance and interventions

**DOI:** 10.1111/dom.70545

**Published:** 2026-02-11

**Authors:** Lanlan Chen, Paul Horn, Frank Tacke

**Affiliations:** ^1^ Department of Hepatology and Gastroenterology Campus Virchow‐Klinikum (CVK) and Campus Charité Mitte (CCM), Charité ‐ Universitätsmedizin Berlin Berlin Germany; ^2^ Department of Hepatobiliary and Pancreatic Surgery General Surgery Center, the First Hospital of Jilin University Changchun China; ^3^ BIH Biomedical Innovation Academy, BIH Charité Digital Clinician Scientist Program Berlin Institute of Health at Charité – Universitätsmedizin Berlin Berlin Germany

**Keywords:** cardiovascular disease, diabetes complications, fatty liver disease, GLP‐1, obesity care, weight management

## Abstract

Metabolic dysfunction‐associated steatotic liver disease (MASLD), characterised by hepatic steatosis and metabolic dysfunction (i.e., obesity, type 2 diabetes, dyslipidaemia, and hypertension), is affecting over 30% of the adult population worldwide. It can progress to metabolic dysfunction‐associated steatohepatitis (MASH) and fibrosis, cirrhosis and even hepatocellular carcinoma. However, most patients with non‐cirrhotic MASLD die from extrahepatic causes, particularly cardiovascular disease and non‐hepatic cancers, underscoring the need for a holistic approach to surveillance and treatment. Emerging research on MASLD has revealed a substantial heterogeneity in the MASLD population, driven by sex‐specific factors, genetic susceptibility, cardiometabolic risk profile, lifestyle and socio‐economic determinants, highlighting the necessity of individualised and holistic management of MASLD patients. Although lifestyle intervention remains the cornerstone of MASLD, the pharmacotherapeutic landscape is rapidly evolving, with resmetirom and semaglutide now approved for non‐cirrhotic MASH with moderate‐to‐advanced fibrosis. In addition, metabolic/bariatric surgery has proven to be a highly effective option for patients with MASH. Given its close association with cardiometabolic and malignant comorbidities, MASLD requires individualised, holistic management integrating hepatic and extrahepatic risks. Multiprofessional care, involving among others behavioural therapists, dieticians and physiotherapists, may improve outcomes of lifestyle interventions, particularly in high‐risk settings. A stepwise and integrated care model combining early case‐finding, risk stratification, and tailored lifestyle and pharmacological interventions is essential to address both hepatic and extrahepatic complications. This review summarises the current understanding of MASLD heterogeneity, clinical assessment, and therapeutic advances, and outlines principles for individualised and coordinated care.

## Plain Language Summary

Metabolic dysfunction‐associated steatotic liver disease (MASLD) is among the most common liver diseases worldwide, affecting over 30% of the adult population. It is characterised by excessive lipid accumulation in the liver along with cardiometabolic risk factors such as obesity, type 2 diabetes, abnormal serum glucose, abnormal serum lipids, or high blood pressure. If left untreated, MASLD can progress to liver inflammation, fibrosis, cirrhosis, and liver cancer. Yet, most people with early MASLD do not die from liver‐related events, but from cardiovascular diseases or cancers outside the liver, showing that MASLD affects the whole body rather than just the liver. MASLD develops differently from person to person, and many factors (e.g., sex, genetics, lifestyle, socio‐economic conditions) play important roles as they impact on the liver, heart, kidney, and other organs. This means that healthcare in individuals with MASLD should be holistic, considering liver disease and extrahepatic risk factors alike. A simple stepwise case‐finding algorithm helps to identify patients at risk in primary care, and appropriate risk stratification can pave the way for individualised management. Healthy lifestyle modification remains the foundation of treatment. Weight loss, regular exercise, improved diet, and objective assessment of alcohol intake are essential. New medications such as resmetirom and semaglutide now offer options for patients with advanced fibrosis. For some individuals with severe MASLD, bariatric surgery can also be effective. This review provides guidance on early detection, risk stratification, and personalised, holistic care to improve long‐term health outcomes in MASLD.

## 1 INTRODUCTION

### 1.1 Metabolic dysfunction‐associated steatotic liver disease

Metabolic dysfunction‐associated steatotic liver disease (MASLD) is defined by the presence of hepatic steatosis with at least one cardiometabolic risk factor, including overweight/obesity, dysglycaemia/type 2 diabetes (T2D), dyslipidaemia or hypertension without excessive alcohol consumption (<20 g/day for women and <30 g/day for men).^1^ Two previous disease definitions (non‐alcoholic fatty liver disease [NAFLD] and metabolic dysfunction‐associated fatty liver disease [MAFLD]) have different limitations, for example, “NAFLD” does not reflect the metabolic characteristics^2^, ^3^ while the word “fatty” in “MAFLD” may be perceived as stigmatising to patients.^1^, ^4^ In Table 1, we compare the three definitions of this disease, and we will adhere to the “MASLD” definition throughout the review. MASLD sits within the broader umbrella of steatotic liver disease (SLD), which encompasses all conditions characterised by hepatic fat accumulation regardless of underlying cause.^1^ In this framework, MASLD is positioned as the most common and metabolically driven component of SLD, capturing individuals in whom metabolic dysfunction plays a central role in the development of hepatic steatosis, fibrosis progression, and associated cardiometabolic risk.

**TABLE 1:** Characteristics of disease definitions based on different nomenclatures.

	NAFLD	MAFLD	MASLD
Full name	Non‐alcoholic fatty liver disease	Metabolic (dysfunction)‐associated fatty liver disease	Metabolic dysfunction‐associated steatotic liver disease
Year introduced	Firstly proposed in 1980^3^; standardised in 2012 from the joint guideline by AASLD, ACG and AGA.^2^	2020^5^	2023^1^
Defining basis	Exclusion of other causes of hepatic steatosis	Positive criteria based on metabolic dysfunction	Positive criteria; unified “steatotic liver disease” framework
Diagnostic criteria	Hepatic steatosis (≥5%) by either imaging or histology; exclusion of secondary causes of liver fat accumulation, for example, significant alcohol intake, viral hepatitis, steatogenic medication, monogenic hereditary steatosis or other secondary causes.	Hepatic steatosis detected by one of imaging, blood biomarkers or histology; At least one of overweight/obesity, type 2 diabetes, or ≥ 2 metabolic risk factors^a^	Hepatic steatosis by imaging or histology; At least one of five cardiometabolic risk factors indicated by overweight or obesity, dysglycaemia or type 2 diabetes, plasma triglycerides, HDL‐cholesterol and blood pressure.
Exclusion of alcohol use in diagnosis	Yes (“significant alcohol consumption” is defined by >21 standard drinks per week in men and >14 standard drinks per week in women and a standard alcoholic drink contains 14 g of pure alcohol)	No (alcohol or viral hepatitis may coexist)	No (if above threshold, classified as MetALD, >30 g/day in men and >20 g/day in women; if alcohol intake >60 g/day in men and >50 g/day in women: ALD)
Key concept	“Nonalcoholic” definition by exclusion	Metabolic dysfunction as a defining feature	Unified classification of steatotic liver disease, recognising metabolic and non‐metabolic causes

^a^ The two risk factors include (adapted from the MAFLD consensus^5^): (1) increased waistline circumference—at least 102 cm for Caucasian men or 88 cm for Caucasian women, or 90 cm for Asian men and 80 cm for Asian women. (2) High blood pressure, defined as 130/85 mmHg or higher, or being on antihypertensive medication. (3) Elevated triglycerides, at or above 150 mg/dL (1.70 mmol/L), or taking triglyceride‐lowering medication. (4) Low HDL cholesterol—below 40 mg/dL (1.0 mmol/L) in men or 50 mg/dL (1.3 mmol/L) in women, or being treated for this issue. (5) Prediabetes, shown by fasting glucose 100–125 mg/dL, a 2‐h glucose level 140–199 mg/dL, or an HbA1c of 5.7–6.4%. (6) Insulin resistance, reflected by a HOMA‐IR score of 2.5 or higher. (7) A raised high‐sensitivity C‐reactive protein (hs‐CRP) level of more than 2 mg/L.

Alcohol consumption, even in moderate amounts, is increasingly recognised as an important risk factor that can synergistically accelerate the progression of MASLD,^6^ while cardiometabolic risk factors accelerate complications in alcohol‐associated liver diseases (ALD).^7^ The new nomenclature introduces “Metabolic Dysfunction and Alcohol‐associated Liver Disease” (MetALD) to specifically classify steatotic liver disease (SLD) in individuals who meet the criteria for MASLD but also consume alcohol above low‐risk thresholds (20–50 g/day for women; 30–60 g/day for men).^8^ This classification highlights the concept of SLD as a disease spectrum where MASLD, ALD, and MetALD represent overlapping categories driven by varying degrees of metabolic dysfunction and alcohol exposure.^9^

**FIGURE 1:**
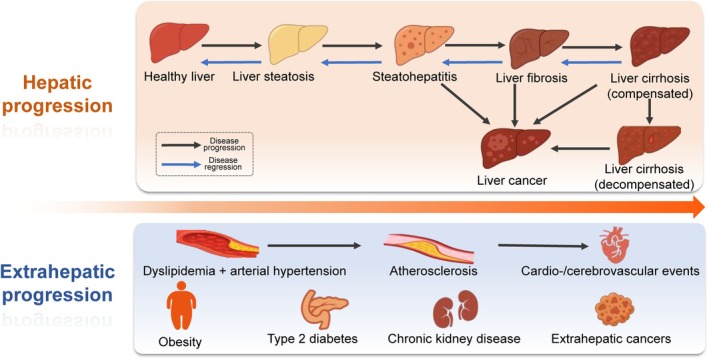
Clinical trajectory of MASLD. MASLD progresses along a continuum from healthy liver to steatosis, MASH, fibrosis, cirrhosis, and HCC. Individuals with MASLD are also at risk for extrahepatic complications, including CVD, CKD, obesity, T2D, and extrahepatic cancers. Disease pathways can diverge towards hepatic progression or systemic metabolic deterioration or even both, influenced by obesity, dyslipidaemia, hypertension, genetics, sex, lifestyle, and socio‐economic determinants. Importantly, MASH resolution and fibrosis regression are possible with sustained lifestyle modification, pharmacological interventions and risk factor management. Even compensated cirrhosis can be regressed. CKD, chronic kidney disease; CVD, cardiovascular disease; HCC, hepatocellular carcinoma; MASH, metabolic dysfunction‐associated steatohepatitis; MASLD, metabolic dysfunction–associated steatotic liver disease; T2D, type 2 diabetes.

The natural history of MASLD disease spectrum follows a trajectory, ranging from simple steatosis, through metabolic dysfunction‐associated steatohepatitis (MASH) and fibrosis, and the ultimate development of liver cirrhosis, which can progress to liver failure or hepatocellular carcinoma (HCC).^10^ Importantly, MASH—particularly in advanced stages of fibrosis—can progress to HCC in the absence of cirrhosis^11^ (Figure 1). Beyond hepatic progression, MASLD is associated with a range of extrahepatic comorbidities, for example, increasing the risk of cardiovascular disease (CVD), chronic kidney disease (CKD) and T2D. Indeed, the majority of patients with non‐cirrhotic MASLD will die from extrahepatic causes, particularly due to CVD and extrahepatic cancers (Figure 1).^5^, ^12^

### 1.2 Global burden and clinical significance

The global prevalence of MASLD is estimated to be 38% in adults and 7%–14% in children^13^ with an estimated 3% to 5% prevalence of MASH.^14^ While isolated hepatic steatosis may not inherently constitute a disease entity (but rather be a metabolic risk indicator), MASH represents a clinically significant disease state with a clear trajectory towards advanced fibrosis and cirrhosis.^15^ From 2021 to 2040, MASH prevalence is projected to rise across major countries, with advanced disease (F3 fibrosis to liver transplantation) increasing by at least 20%. Consequently, the direct annual medical costs are expected to more than double, alongside substantial productivity losses and modest declines in health‐related quality of life.^16^ Clinically, MASLD carries a burden of both hepatic risks (major adverse liver outcomes [MALO]) and extrahepatic comorbidities (obesity, T2D, CVD, CKD, and extrahepatic cancers).^14^ MALO includes the development of cirrhosis, hepatic decompensation, HCC, liver transplantation, and liver‐related death, serving as key endpoints of advanced liver disease progression. In addition to MALO, CVD is one of the major extrahepatic causes of death in non‐cirrhotic MASLD patients as the cardiac‐specific mortality accounts for nearly half of all‐cause mortality, highlighting the significance of a holistic approach to the management of MASLD.^14^

## 2 RISK PROFILES AND HETEROGENEITY OF MASLD

### 2.1 Sex‐specific characteristics of MASLD and other factors affecting heterogeneity

Growing evidence highlights the pivotal role of sex as a biological variable in MASLD, affecting distinct susceptibility, hormonal factors, and disease trajectories in males and females.^17^, ^18^ This emerging topic is important as the susceptibility to MASLD, as well as its progression and regression is different between males and females.^19^, ^20^, ^21^ The prevalence of MASLD is substantially lower in premenopausal women (9% to 18%) compared to men (20.2% to 41%). However, this changes with menopause, after which the prevalence increases to over 30% in women, indicating that postmenopausal women constitute a risk group that requires particular medical attention.^20^ These sex differences are likely largely determined by sex hormone effects. Oestrogen can improve insulin sensitivity as well as hepatic function, thereby protecting premenopausal women against MASLD; in fact, the risk increase following menopause may be partly explained by decreases in oestrogen levels.^20^ Besides sex hormones, the sex chromosomes are also critical in the pathogenesis as they can impact adiposity (e.g., the dosage of KDM5C, an X chromosome gene, can affect adiposity and lead to sex differences)^22^; however, more evidence is expected in the future.^23^, ^24^

**FIGURE 2:**
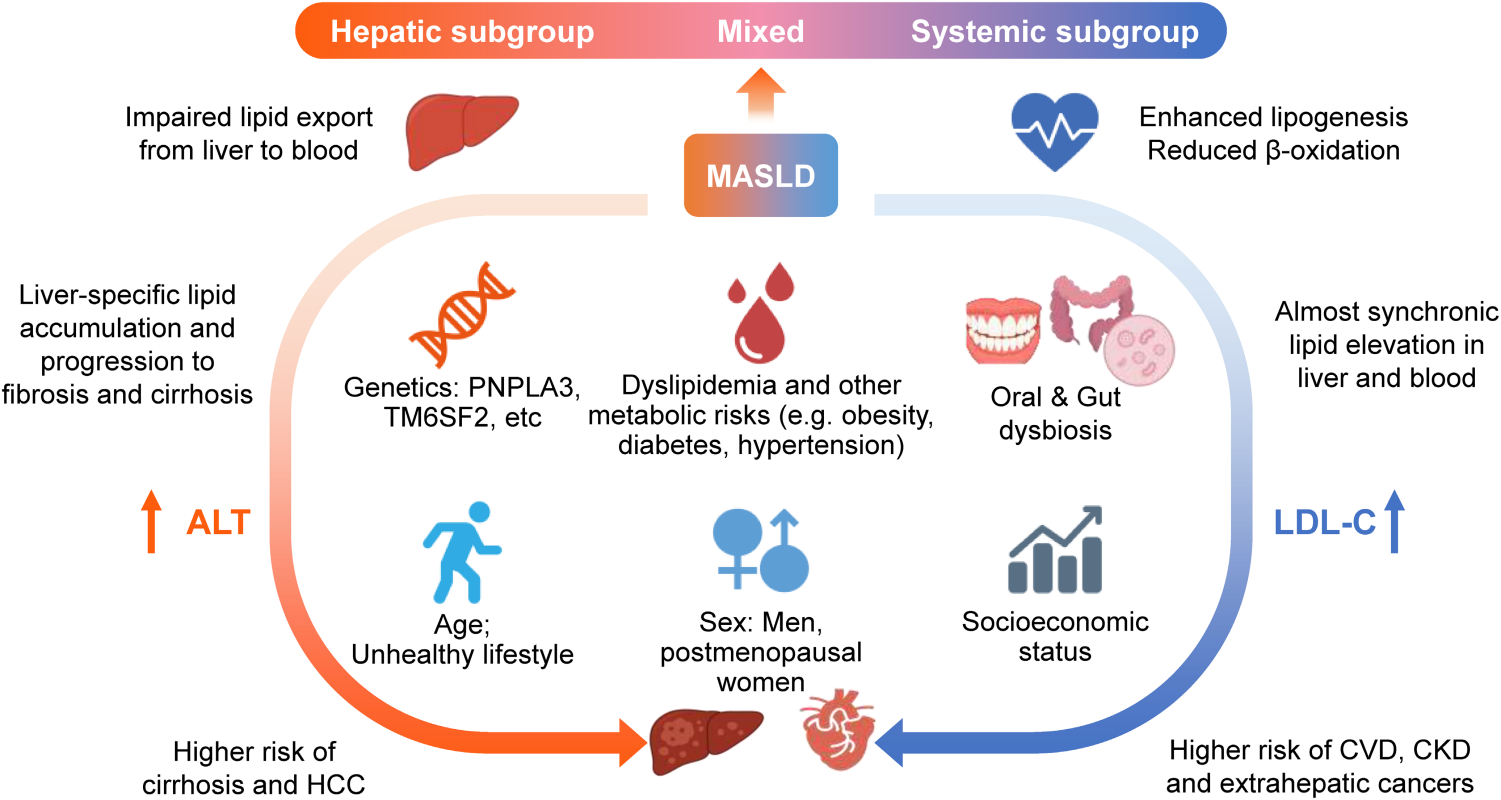
Heterogeneity and sex‐specific, genetic, and metabolic profiles in MASLD. MASLD encompasses diverse metabolic phenotypes influenced by genetic variants (e.g., PNPLA3, TM6SF2), gut dysbiosis, lipid metabolism abnormalities, lifestyle factors, age, and socio‐economic conditions. Distinct subtypes include a hepatic‐predominant pattern, characterised by impaired hepatic lipid export and higher risk of fibrosis and HCC, and a systemic‐predominant pattern, marked by enhanced lipogenesis and increased circulating lipids with higher CVD risk. Sex differences further shape susceptibility and progression: men generally carry a higher risk, whereas postmenopausal women show increased vulnerability. ALT, alanine aminotransferase; CVD, cardiovascular disease; HCC, hepatocellular carcinoma; LDL‐C, low‐density lipoprotein cholesterol; MASLD, metabolic dysfunction‐associated steatotic liver disease; PNPLA3, patatin‐like phospholipase domain–containing protein 3; TM6SF2, transmembrane 6 superfamily member 2.

In addition to sex, other factors such as nutrition, alcohol consumption, oral hygiene, lifestyle behaviours, age, and socio‐economic status influence MASLD susceptibility and progression largely through their effects on obesity and other domains of metabolic dysfunction (i.e., insulin resistance), which particularly interact with alcohol and genetic predisposition to shape disease trajectories in MASLD (Figure 2). Dietary composition and caloric intake directly fuel hepatic lipid accumulation and insulin resistance, while even low‐to‐moderate alcohol consumption synergises with metabolic stress to exacerbate liver injury and accelerate fibrosis progression in MASLD.^6^, ^25^ Physical inactivity and a sedentary lifestyle are additional risk factors, whereas regular exercise confers metabolic and hepatic health benefits.^25^ Age‐related changes in metabolism, hormonal status, and adipose tissue distribution further modify disease risk as age‐related declines in mitochondrial efficiency, hormonal shifts (such as reduced oestrogen in women or reduced testosterone in men), and increased visceral fat collectively worsen insulin resistance and raise hepatic lipid influx, thereby increasing the risk and severity of MASLD.^26^, ^27^ Visceral adiposity in particular drives hepatic delivery of free fatty acids and inflammatory signalling via the portal vein, further promoting hepatic steatosis and metabolic dysfunction.^28^, ^29^ Furthermore, recent data demonstrated a connection between dental hygiene, periodontal diseases and MASLD, as oral dysbiosis can accelerate the progression of MASLD; and advanced fibrosis and cirrhosis are associated with a higher risk of periodontal diseases.^30^ Last but not least, socio‐economic factors, including education, income, and access to healthcare and nutritious food, are increasingly recognised as determinants of MASLD prevalence and outcomes.^31^

### 2.2 Heterogeneity of MASLD and potential subgroups

MASLD is a heterogeneous condition as it encompasses diverse genetic susceptibility, nutrition and metabolic profiles, pathophysiological mechanisms, and clinical outcomes. The degree and combination of metabolic syndrome traits, ranging from a single factor to all five (i.e., central obesity, raised fasting triglyceride or glucose concentration [or prediabetes/T2D], reduced serum HDL‐C and elevated blood pressure), determine variable risks of hepatic and extrahepatic complications.^32^ In a Swedish cohort of 10 568 individuals with MASLD, deaths were mainly due to extrahepatic cancers and liver cirrhosis rather than CVD.^33^ However, a smaller biopsy‐based cohort of 959 patients showed stage‐dependent differences: liver disease was the leading cause of death in those with cirrhosis, whereas CVD and extrahepatic cancers predominated in non‐cirrhotic fibrosis.^34^ A Chinese cohort implicated that fibrotic MASLD patients with normal ALT are less responsive to lifestyle intervention compared to those with elevated ALT, highlighting the heterogeneity and need for a tailored therapy for MASLD.^35^ Beyond metabolic traits, genetic variants (e.g., *PNPLA3* and *TM6SF2*), gut microbiota alterations, bile acid dysregulation, and inter‐organ crosstalk involving the liver, adipose tissue, heart, and kidneys contribute to further heterogeneity.^32^, ^36^

Recent studies unveiled at least two subtypes of MASLD that differ regarding hepatic progression versus systemic progression: (i) the first study classified MASLD based on polygenic risk scores^37^; (ii) the second study employed six clinical parameters including age, BMI, ALT, HbA1c, LDL‐C, and triglycerides (Figure 2).^38^ Both MASLD subtypes are closely associated with T2D while the hepatic subtype carries a higher risk to develop cirrhosis and HCC, and the systemic subtype carries a higher risk of CVD. The hepatic subgroup was mainly characterised by hepatic lipid accumulation due to impaired hepatic triglyceride export,^37^, ^39^ suggesting direct hepatocellular lipotoxicity with significantly increased ALT levels (Figure 2).^38^ However, the systemic subgroup demonstrated increased de novo lipogenesis and impaired lipid oxidation, and the function of lipid transport remains intact,^37^, ^39^ leading to increased serum levels of triglycerides and LDL‐C (Figure 2).^38^ These two subgroups can, to some extent, explain why some MASLD patients are at higher risk for MALO (probably the hepatic subgroup) while others primarily develop CVD (probably the systemic subgroup).

## 3 KEY ASPECTS OF CLINICAL ASSESSMENT

### 3.1 MASLD case‐finding in routine clinical care

**FIGURE 3:**
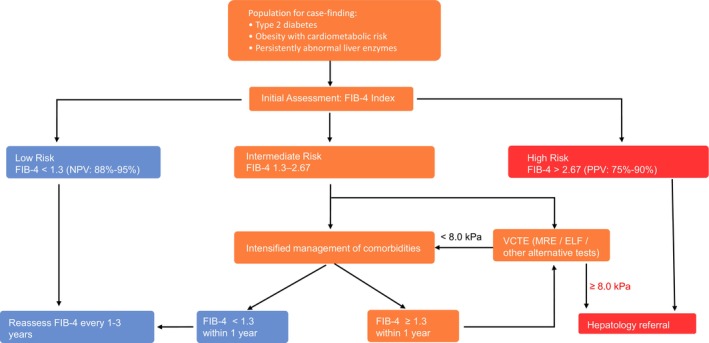
Stepwise algorithm for case‐finding and risk stratification of advanced liver fibrosis using the FIB‐4 index in high‐risk populations (partially adapted from the EASL guideline). Individuals with type 2 diabetes, obesity with cardiometabolic risk, or persistently abnormal liver enzymes undergo initial non‐invasive assessment with the FIB‐4 index. A FIB‐4 value <1.3 indicates low risk for advanced fibrosis, with a high negative predictive value (NPV ~88%–95%), and periodic reassessment every 1–3 years is recommended. Patients with intermediate risk (FIB‐4 1.3–2.67) should receive intensified management of metabolic comorbidities and repeat FIB‐4 assessment within 1 year. If FIB‐4 remains ≥1.3, second‐line fibrosis assessment with vibration‐controlled transient elastography (VCTE) or alternative elastography‐based methods (e.g., MRE, ELF) is advised. Liver stiffness <8.0 kPa supports continued non‐invasive follow‐up, whereas values ≥8.0 kPa prompt referral to hepatology. A FIB‐4 value >2.67 identifies high‐risk individuals with a high positive predictive value (PPV ~75%–90%) for advanced fibrosis, for whom direct referral to specialist care is recommended. EASL, European Association for the Study of the Liver; ELF, Enhanced Liver Fibrosis (test); FIB‐4, Fibrosis‐4 index; MASLD, metabolic dysfunction‐associated steatotic liver disease; MRE, magnetic resonance elastography; NAFLD, non‐alcoholic fatty liver disease; NPV, negative predictive value; PPV, positive predictive value; VCTE, vibration‐controlled transient elastography.

Early identification of individuals with “at‐risk” MASLD/MASH and clinically significant fibrosis (MASH fibrosis) is a critical unmet need, as these patients carry the highest risk for liver‐related complications and mortality (Figure 3). Because most affected individuals remain asymptomatic, structured case‐finding strategies in routine clinical care are required to detect those at risk before progression to advanced disease occurs. Rather than universal screening, European Clinical Practice Guidelines advocate an active case‐finding approach in populations at highest risk of MASLD with fibrosis^4^ (Figure 3). Healthcare providers, particularly primary care providers, should actively look for “at‐risk” MASLD/MASH in individuals with either T2D, obesity with additional cardiometabolic risk factors/metabolic syndrome, or in those presenting with persistently elevated liver enzymes or liver steatosis in imaging studies.^4^, ^40^ Besides, other familial risks (e.g., family history of cirrhosis, premature CVD, T2D), polycystic ovarian syndrome (PCOS), hypothyroidism, socio‐economic status, and lifestyle patterns (e.g., poor diet quality, physical inactivity/sedentary time, sleep disturbance/obstructive sleep apnea, and alcohol intake) may also be considered in guiding case finding.^4^, ^41^

### 3.2 Laboratory, imaging, and AI‐based evaluation in MASLD

Routine biochemical tests (i.e., ALT, AST, ALP, GGT, and albumin) remain essential parameters in the first‐line assessment for suspected MASLD. Elevated aminotransferases above revised sex‐specific thresholds (ALT >33 U/L in men and >25 U/L in women) indicate hepatocellular injury but are neither sensitive nor specific for the presence or severity of the disease.^40^ Importantly, normal enzyme levels do not exclude significant fibrosis or steatohepatitis. Still, persistently abnormal liver tests should prompt diagnostic evaluation to exclude other concomitant liver diseases such as viral hepatitis or autoimmune liver disease.^4^

**FIGURE 4:**
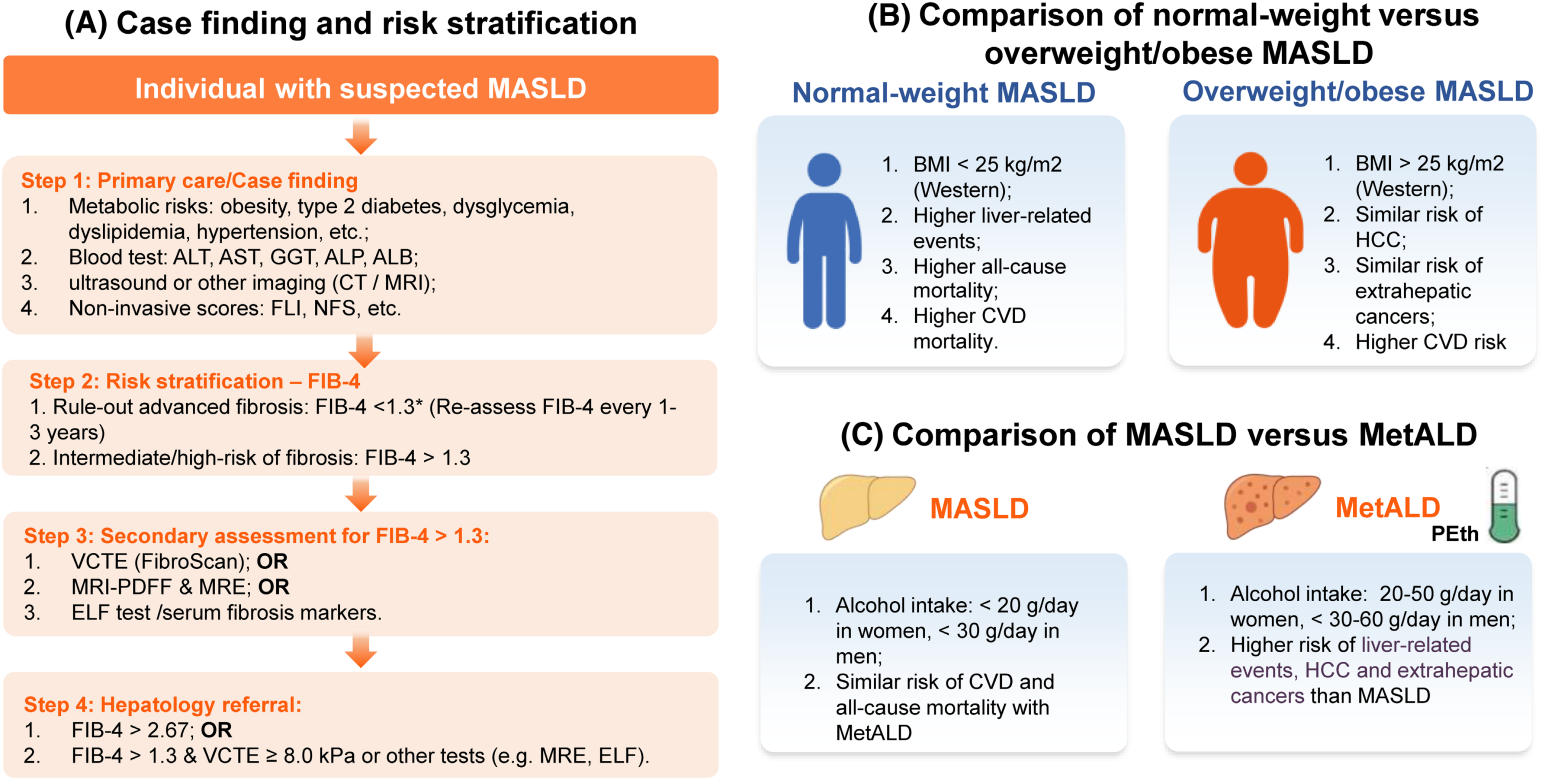
Key clinical assessment of MASLD. (A) Case finding and risk stratification: initial evaluation of individuals with metabolic risk factors includes liver enzymes, metabolic profiling, and non‐invasive fibrosis scoring (e.g., FIB‐4), followed by VCTE or serum fibrosis markers for indeterminate or high‐risk cases. This stepwise approach supports early identification of individuals at risk for advanced fibrosis. (B) Comparison of normal‐weight versus overweight/obese MASLD: normal‐weight MASLD (normal BMI) and overweight/obese MASLD present distinct metabolic and clinical characteristics. Normal‐weight MASLD is associated with higher risks of liver‐related events, CVD mortality and all‐cause mortality, whereas overweight/obese MASLD is more strongly linked to systemic metabolic dysfunction. Recognition of these phenotypic differences is critical for accurate diagnosis, metabolic evaluation, and individualised management. (C) Comparison of MASLD and MetALD: MASLD (no/low alcohol intake) and MetALD (metabolic dysfunction plus moderate alcohol consumption) share overlapping features but differ in liver‐related risk. MetALD is associated with higher rates of liver‐related events, HCC and extrahepatic cancers. Objective alcohol assessment, including structured questionnaires and biomarkers such as PEth, is essential for accurate classification and appropriate therapeutic decisions. *The FIB‐4 cut‐off is age‐adjusted for age >65 years. ALP, alkaline phosphatase; ALT, alanine aminotransferase; AST, aspartate aminotransferase; BMI, body mass index; CVD, cardiovascular disease; CT, computed tomography; ELF, Enhanced Liver Fibrosis test; FIB‐4, fibrosis‐4 index; FLI, fatty liver index; GGT, γ‐glutamyltransferase; HCC, hepatocellular carcinoma; MASLD, metabolic dysfunction–associated steatotic liver disease; MetALD, metabolic and alcohol‐associated liver disease; MRE, magnetic resonance elastography; MRI‐PDFF, magnetic resonance imaging–proton density fat fraction; NFS, NAFLD fibrosis score; PEth, phosphatidylethanol; VCTE, vibration‐controlled transient elastography.

Risk stratification strategies are mainly based on non‐invasive tools for assessing liver fibrosis. International guidelines suggest initiating evaluation with widely available blood‐based fibrosis scores, such as the FIB‐4, which combines age, AST, ALT, and platelet count.^4^, ^40^ A step‐wise approach is recommended, where individuals with indeterminate or high fibrosis risk (i.e. FIB‐4 >1.3 in individuals ≤65 years of age, FIB‐4 >2.0 in individuals >65 years) proceed to second‐line imaging testing with vibration‐controlled transient elastography (VCTE) or serum fibrosis markers such as enhanced liver fibrosis (ELF) test (Figures 3, 4A).^4^, ^40^ Patients with indeterminate or discordant results, or with advanced disease on non‐invasive testing, are candidates for hepatology referral and further diagnostic workup, which may still include liver biopsy in selected cases.^4^, ^40^ Liver biopsy remains the gold standard for diagnosing MASH and staging fibrosis, but its role is now primarily confirmatory when non‐invasive methods yield inconclusive or atypical results. Histology retains value for identifying overlapping aetiologies, including autoimmune, drug‐induced, or genetic liver injury.^4^

Other imaging modalities provide complementary insights into liver disease evaluation (Figure 4A): (a) ultrasound is the most widely available and cost‐effective tool for detecting hepatic steatosis but lacks sensitivity for mild steatosis and cannot reliably assess fibrosis. (b) Magnetic resonance imaging‐proton density fat fraction (MRI‐PDFF) provides accurate and reproducible quantification of hepatic fat content and is increasingly used to monitor therapeutic responses in clinical trials but is not widely available in clinical practice. (c) Magnetic resonance elastography (MRE) offers the highest diagnostic accuracy for assessing fibrosis across aetiologies among imaging modalities but, as MRI‐PDFF, remains limited by high cost, availability, and technical complexity. The integration of these imaging approaches facilitates longitudinal monitoring and characterisation of phenotypic heterogeneity, although selection should be tailored to available resources and the specific healthcare setting (i.e., primary, secondary, or tertiary care).

Emerging artificial intelligence (AI)‐based approaches are likely to transform MASLD evaluation and risk stratification. Large language models (LLMs) and multimodal AI systems can integrate electronic health records and ultrasound or histology images to diagnose MASLD with accuracy comparable to traditional indices such as the fatty liver index (FLI).^42^ Advanced generative AI frameworks, such as retrieval‐augmented LLMs, can support clinical decision‐making by combining guideline‐based reasoning with patient‐specific data, thereby reducing diagnostic variability and supporting real‐time fibrosis risk estimation.^43^ Furthermore, AI‐derived fibrosis scores FIB‐9, FIB‐11, and especially FIB‐12, which were built using multitargeted machine‐learning, integrating routine and specialised blood markers as well as liver stiffness markers, substantially improve non‐invasive detection of advanced fibrosis in MASLD, with FIB‐12 outperforming all existing tests.^44^ However, further prospective validation in diverse populations is needed before these models may be implemented in clinical practice.

### 3.3 Normal‐weight MASLD: A special subgroup with unique risks

Normal‐weight MASLD refers to MASLD in individuals with BMI <25 kg/m^2^ (Western) or <23 kg/m^2^ (Asian), and its prevalence may be about 13% of the global population, accounting for 10%–30% of patients with MASLD.^45^ Contrary to traditional concepts, normal‐weight MASLD is not a benign phenotype.^46^ In a recent large‐scale multicohort study including >180 000 participants, normal‐weight MASLD was associated with higher risks of liver‐related events, CVD mortality and all‐cause mortality, alongside a similar risk for HCC and extrahepatic cancers compared with overweight/obese MASLD (Figure 4B).^47^, ^48^, ^49^ It should be noted that “normal‐weight” MASLD defined by BMI may be associated with increased visceral or ectopic adiposity, as assessed by waist circumference or imaging modalities, and this phenotype may carry equal or even higher CVD mortality compared with obese MASLD. Since normal‐weight MASLD is frequently under‐recognised because of its normal BMI, thereby delaying diagnosis and care, early case‐finding approaches should rely on metabolic risk profiling and non‐invasive fibrosis assessment (e.g., FIB‐4, VCTE) rather than BMI alone.^45^ Management should emphasise moderate weight loss, aiming to improve metabolic status and preserve muscle mass. Attention should also be put on the detection of potential underreported alcohol consumption (metabolic and alcohol‐associated liver disease [MetALD]) and genetic predisposition.^45^

### 3.4 Discriminating MASLD from MetALD: Similar diseases with different outcomes

MetALD is defined as steatotic liver disease in patients who meet cardiometabolic criteria for MASLD and consume alcohol above consensus “moderate” thresholds (i.e., >20 g/day for women or >30 g/day for men), but yet below the amounts that define alcohol‐related liver disease (ALD; >50 g/day for women, >60 g/day for men), separating MetALD from both MASLD (no/low alcohol intake) and ALD (high alcohol intake irrespective of metabolic risk).^50^ In US population‐based data from NHANES 2017–2023 using transient elastography, the age‐adjusted prevalence estimates were about 31.9% for MASLD and about 2.2% for MetALD, quantifying MetALD as a smaller but non‐trivial slice of the SLD spectrum.^51^ Of note, SLD classifications are highly dynamic instead of fixed entities. Over a 2‐year period as many as 38% of individuals shifted categories, mainly due to changes in reported alcohol intake or no longer meeting metabolic criteria.^52^ This diagnostic fluidity supports the need for periodic reassessment of alcohol exposure and cardiometabolic risk, both for clinical follow‐up and trial eligibility considerations.

A systematic review and meta‐analysis directly compared MASLD with MetALD and found that MetALD carries higher risks of liver‐related events, hepatocellular carcinoma, and extrahepatic cancers than MASLD, while cardiovascular event rates and all‐cause mortality were similar (Figure 4C).^53^ These results support that the management of cardiometabolic and other extrahepatic risks remains essential for MetALD; specifically, MetALD patients may need more intensive liver‐focused surveillance, particularly for HCC.^53^

### 3.5 Objective assessment of alcohol intake in clinical practice

Accurate quantification of alcohol intake is essential for distinguishing MASLD from MetALD. A 2025 Delphi consensus emphasised the integration of objective biomarkers with standardised self‐reporting tools to improve diagnostic precision, outlining a structured approach for assessing alcohol consumption in steatotic liver disease (SLD).^54^ It recommends that alcohol intake should be expressed in grams per week using locally defined standard drink conversions and that all patients undergo screening with Alcohol Use Disorders Identification Test (AUDIT) or AUDIT‐consumption (AUDIT‐C) questionnaires for alcohol use disorder (AUD).^55^ When self‐reported alcohol use is uncertain or inconsistent, direct biomarkers (particularly phosphatidylethanol [PEth] in blood; ethyl glucuronide [EtG] in hair or urine) should be incorporated. PEth, a blood‐based biomarker unaffected by sex or BMI, reflects alcohol intake during the preceding 1–3 weeks; levels <20 ng/mL rule out relevant alcohol use, 20–200 ng/mL suggest moderate use (consistent with MetALD), and ≥200 ng/mL indicate harmful consumption.^56^ These biomarkers outperform questionnaire screening in sensitivity for recent drinking and help identify patients whose reported intake lies in the diagnostic “border zone” between MASLD and MetALD.^54^ A crucial consideration in the interpretation of PEth testing for chronic excessive alcohol consumption is the potential for false‐positive results due to confounders such as recent blood transfusions or the use of ethanol‐containing medications, as these can introduce PEth precursors into the bloodstream.^57^ The consensus further advises reassessing metabolic and hepatic status after 8–12 weeks of abstinence or reduced drinking to determine whether alcohol or metabolic dysfunction is the predominant driver of disease.^54^ In summary, a dual strategy combining validated questionnaires and direct biomarkers, especially PEth, provides an evidence‐based, objective framework for quantifying alcohol exposure in clinical and research settings for SLD.

### 3.6 Cardiometabolic and other extrahepatic risk evaluation

#### 3.6.1 CVD risk, dyslipidemia, and hypertension

People with MASLD have consistently higher rates of major adverse cardiovascular events compared to the general population; and CVD is the leading cause of death in the non‐cirrhotic MASLD population. Accordingly, every patient should receive a formal 10‐year CVD risk estimation, including a structured review of blood pressure, lipid profile, smoking status, and pre‐existing CVD.^58^ Beyond dyslipidaemia, hypertension deserves particular attention: it is common, frequently uncontrolled, and is linked not only to CVD risk but also to faster liver stiffness/fibrosis progression and worse long‐term outcomes, supporting early detection and guideline‐directed therapy.^59^, ^60^ These steps fit an emerging “heart–liver” co‐management model that integrates non‐invasive liver fibrosis assessment into cardiovascular care and routine blood pressure/lipid management into hepatology workflows.^59^

#### 3.6.2 Diabetes and insulin resistance

Insulin resistance is central to MASLD pathophysiology and links hepatic steatosis with atherogenic dyslipidaemia and systemic inflammation. Systematic screening for hyperglycaemia (fasting glucose/HbA1c) is warranted, and coexisting T2D substantially amplifies both macro‐ and microvascular risks, together with malignancies.^58^

#### 3.6.3 Renal function and CKD risk

CKD and MASLD share risk factors (e.g., obesity, hypertension, dyslipidaemia, insulin resistance).^61^ A meta‐analysis shows higher CKD prevalence and incidence in MASLD, with stronger associations in patients with steatohepatitis and/or advanced fibrosis.^61^, ^62^ Therefore, patients diagnosed with MASLD should be evaluated for CKD by assessing estimated glomerular filtration rate (eGFR) and albuminuria at baseline and periodically during follow‐up.

#### 3.6.4 Malignancy: Hepatocellular and extra‐hepatic cancers

Importantly, extra‐hepatic cancers (notably colorectal, breast, uterine, prostate, lung, kidney) are more frequent overall than HCC in MASLD and are not confined to advanced liver disease.^5^ Until MASLD‐specific risk tools for extrahepatic cancer development are validated, patients should participate in standard recommended population screening programs (e.g., colorectal, breast, cervical, prostate per local policy) regardless of fibrosis stage.^5^

The risk for HCC development in MASLD rises sharply with advanced fibrosis/cirrhosis,^63^ and all patients with cirrhosis should undergo biannual screening by ultrasound, optionally complemented by assessment of alpha fetoprotein (AFP).^4^ Still, a meaningful fraction of MASLD‐related HCC occurs without cirrhosis, and cost‐effective surveillance criteria for non‐cirrhotic MASLD remain unsettled.^5^, ^64^ HCC surveillance may be considered in patients with MASH and advanced fibrosis who have additional risk factors (e.g., age, active smoking, long history of obesity and T2D), or in whom the annual HCC risk exceeds 1.5% according to validated risk scores such as aMAP.^65^

## 4 INDIVIDUALISED CARE PLANS

### 4.1 Lifestyle intervention tailoring

**FIGURE 5:**
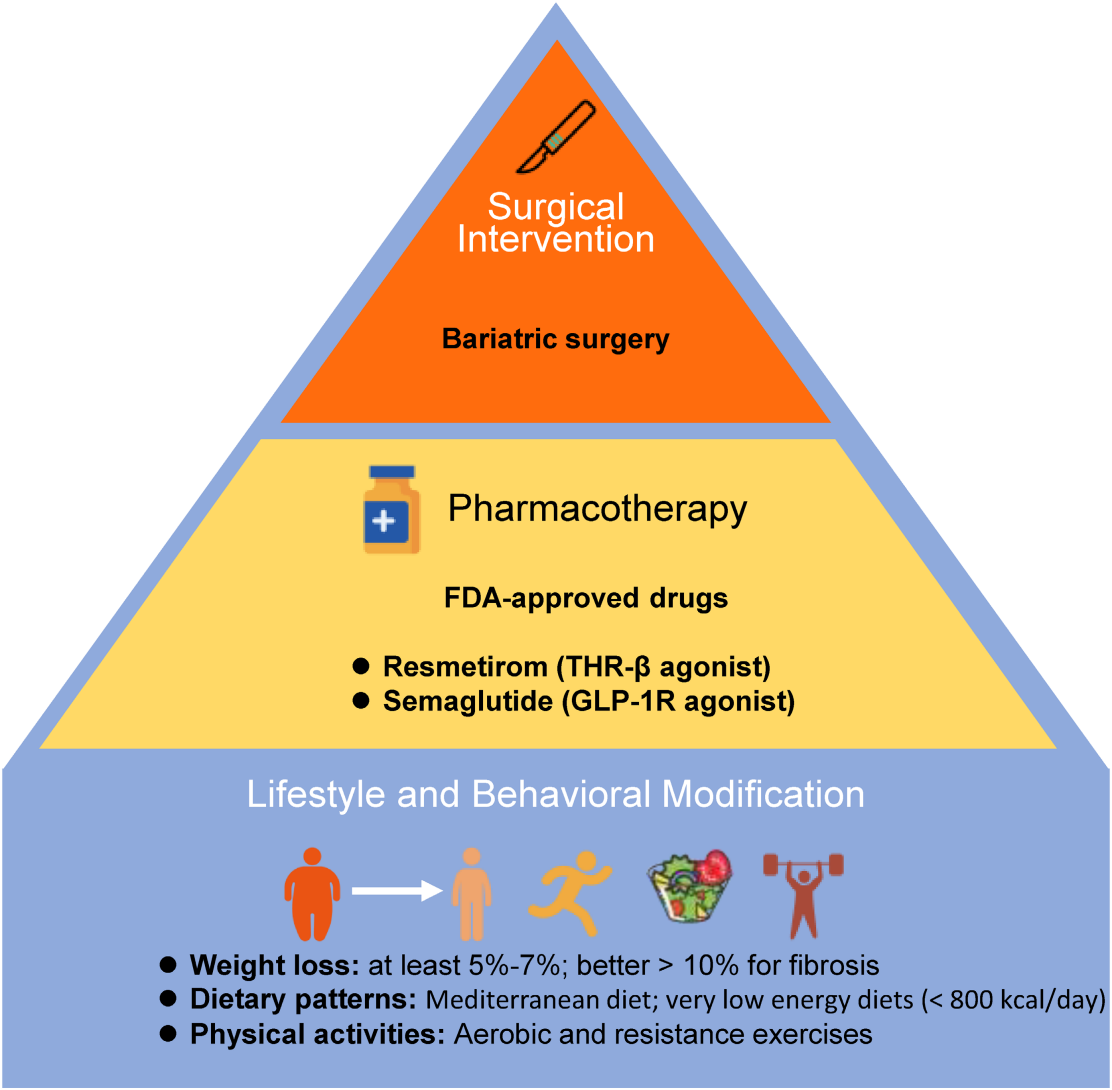
Strategies for individualised care model. The figure outlines the guiding principles of personalised MASLD management. Care plans emphasise personalised goal‐setting and consideration of access to healthy nutrition, physical activity, and medical therapies. GLP‐1R, glucagon‐like peptide‐1 receptor; MASLD, metabolic dysfunction–associated steatotic liver disease; THR‐β, thyroid hormone receptor beta.

Lifestyle modification remains the cornerstone of MASLD management across disease stages (Figure 5). Individualisation should account for but not be limited to genetic susceptibility, sex disparities, socio‐economic status, physical and mental capabilities, and comorbid conditions. Several practical lifestyle interventions are recommended: (a) weight loss: a reduction of ≥10% total body weight induces MASH resolution and fibrosis regression; even 5%–7% loss improves steatosis. Caloric restriction is essential to achieve weight loss; however, calorie goals and weight loss strategies must be individualised according to the degree of obesity, health status as well as physical and mental capabilities. (b) Dietary patterns: diets based on the Mediterranean diet remain the dietary model with highest evidence.^66^ Rapid and meaningful weight loss can be achieved with low‐energy diets, including very low‐energy diets (VLEDs, <800 kcal/day) and severe energy‐reduced diets (SERDs, a ≥65% reduction in energy intake relative to weight‐maintenance requirements), which can result in 10%–15% weight loss within 3 months.^67^, ^68^, ^69^ Both VLEDs and SERDs should only be considered as time‐limited interventions under medical supervision, with careful attention to nutritional adequacy and safety. The diet management also includes the avoidance of alcohol and sugar‐sweetened beverages as well as avoidance of highly processed foods. Consideration of socioeconomic context remains important, as barriers to healthy eating may extend beyond food cost alone. Factors such as time constraints, psychosocial stress, limited meal planning, and inconsistent access to healthy foods at home, which are more prevalent in lower socio‐economic settings or college students, can substantially impair adherence to dietary recommendations and adversely affect metabolic outcomes.^70^, ^71^ These practical challenges should be recognised and addressed when delivering lifestyle advice. (c) Physical activity: aerobic and resistance exercises should be prescribed according to physical capacity and comorbidity profile (preferably moderate physical activity >150 min/week or >75 min/week of vigorous‐intensity physical activity),^4^ improving hepatic steatosis and cardiometabolic health even in the absence of significant weight loss and it can even help to prevent weight regain following pharmacological weight loss interventions.^72^

### 4.2 Pharmacological therapies

Pharmacological therapy should complement, not replace, lifestyle interventions.^4^ Two agents have recently received accelerated FDA approval for MASH (Figure 5): (i) resmetirom, a selective thyroid hormone receptor‐β (THRβ) agonist, indicated for non‐cirrhotic MASH with moderate‐to‐advanced fibrosis (F2–F3), in combination with diet and exercise. It is a liver‐targeted therapy, achieving 25.9%–29.9% MASH resolution and 24.2%–25.9% fibrosis regression at 52 weeks, compared to 9.7% and 14.2%, respectively, with placebo.^73^ The liver‐directed activity may make resmetirom particularly suited to patients with a predominantly hepatic phenotype. Common adverse effects include gastrointestinal symptoms (diarrhoea, nausea), and transient increases in liver enzymes. (ii) semaglutide—a glucagon‐like peptide‐1 (GLP‐1) receptor agonist approved for MASH with moderate‐to‐advanced fibrosis (F2‐F3), acting systemically through weight loss and metabolic improvement.^74^ In the pivotal phase 3 trial, it achieved 62.9% MASH resolution and 36.8% fibrosis improvement at 72 weeks, compared to 34.3% and 22.4% with placebo, respectively.^74^ Side effects largely mirror those of other GLP‐1 RAs, including nausea, vomiting, delayed gastric emptying, and potential risk of gallbladder disease due to rapid weight loss. Given its systemic metabolic benefits, semaglutide may be especially advantageous in patients with obesity, diabetes, or a cardiometabolic MASLD phenotype. Other promising agents for MASH‐directed therapy include the dual GLP‐1/glucose‐dependent insulinotropic polypeptide (GIP) receptor agonist (tirzepatide),^75^ GLP‐1/glucagon receptor co‐agonists (e.g., survodutide),^76^ sodium‐glucose cotransporter 2 (SGLT2) inhibitors such as dapaglifozin^77^ and fibroblast growth factor 21 (FGF21) analogues such as efruxifermin, all currently in late‐phase clinical development^78^ (Table 2).

**TABLE 2:** Selected clinical trials of MASLD with reported results in recent years (between 2023 and 2025).

Class	Agent	Phase (year)	Population	Dose/regimen	Sample size (per arm)	Primary endpoints	Key outcomes	Ref.
GLP‐1RA	Semaglutide 2.4 mg (weekly)	3 (2025)	Biopsy‐confirmed MASH with F2 to F3	2.4 mg SC QW versus placebo for over 72 weeks	2.4 mg *n* = 798; placebo *n* = 399 (2:1; total 1197)	MASH resolution without worsening of fibrosis; fibrosis reduction without worsening of steatohepatitis	MASH resolution: 62.9% semaglutide versus 34.3% placebo; fibrosis improvement: 36.8% semaglutide versus 22.4% placebo	PMID: 40305708^69^
GIP/GLP‐1 dual agonist	Tirzepatide	2b (2024)	Biopsy‐confirmed MASH with F2 to F3	5, 10, 15 mg SC QW versus Placebo for 52 weeks	5 mg *n* = 47; 10 mg *n* = 47; 15 mg *n* = 48; Placebo *n* = 48 (total *n* = 190)	Resolution of MASH without worsening of fibrosis	MASH resolution: 44%–62% across doses versus 10% placebo; fibrosis improvement 51%–55% versus 30% placebo	PMID: 38856224^70^
GLP‐1/Glucagon dual agonist	Survodutide	2b (2024)	Biopsy‐confirmed MASH with F1 to F3	2.4, 4.8, 6.0 mg SC QW versus placebo for 48 weeks	2.4 mg *n* = 73; 4.8 mg *n* = 72; 6.0 mg *n* = 74; placebo *n* = 74 (total *n* = 293)	Histological improvement in MASH without worsening of fibrosis	MASH reduction: 43%–64% across doses versus 14% placebo	PMID: 38847460^71^
GLP‐1/Glucagon dual agonist	Pemvidutide	2b (2025)	Biopsy‐confirmed MASH with F2 to F3	1.2, 1.8 mg SC QW versus placebo for 48 weeks	1.2 mg *n* = 41; 1.8 mg *n* = 85; placebo *n* = 86 (total *n* = 212)	MASH resolution without worsening of fibrosis or at least one stage liver fibrosis improvement without worsening of MASH	MASH resolution: 52%–58% across doses versus 20% placebo. The goal of fibrosis improvement was not met (33%–36% vs. 28% placebo).	PMID: 41237796^75^
THR‐β agonist	Resmetirom	3 (2024)	Biopsy‐confirmed MASH with F1B to F3	80 mg QD; 100 mg QD versus placebo for 52 weeks	80 mg *n* = 322; 100 mg *n* = 323; placebo *n* = 321 (total *n* = 966)	MASH resolution without worsening of fibrosis; fibrosis improvement ≥1 stage without worsening of the NAS.	MASH resolution: 25.9%–29.9% across doses versus 9.7% placebo; fibrosis improvement: 24.2%–25.9% across doses versus 14.2% placebo	PMID: 38324483^68^
FGF21 analogue	Pegozafermin	2b (2023)	Biopsy‐confirmed MASH with F2 to F3	15 mg QW; 30 mg QW; 44 mg Q2W; placebo (QW + Q2W pooled) for 24 weeks	15 mg *n* = 21; 30 mg *n* = 73; 44 mg *n* = 57; placebo *n* = 71 (total *n* = 222)	Fibrosis improvement ≥1 stage without worsening of MASH; MASH resolution without worsening fibrosis	Fibrosis improvement: 22%–27% across doses versus 7% placebo; MASH resolution 23%–37% across doses versus 2% placebo	PMID: 37356033^76^
FGF21 analogue	Efruxifermin	2b (2023)	Biopsy‐confirmed MASH with F2 to F3	28 mg QW; 50 mg QW versus placebo for 24 weeks	28 mg *n* = 42; 50 mg *n* = 43; placebo *n* = 43 (total *n* = 128)	Fibrosis improvement ≥1 stage without worsening of MASH	Fibrosis improvement: 39%–41% versus 20% placebo; MASH resolution improved	PMID: 37802088^77^
SGLT2 inhibitor	Dapagliflozin	2 (2025)	Biopsy‐confirmed MASH with/without T2D	10 mg PO QD versus placebo for 48 weeks	10 mg *n* = 78; placebo *n* = 76 (total *n* = 154)	MASH improvement (NAS reduction ≥2 or NAS ≤3) without worsening fibrosis	Primary met: 53% versus 30%; MASH resolution 23% versus 8%; fibrosis improvement 45% versus 20%	PMID: 40467095^72^
FASN inhibitor	Denifanstat	2b	Biopsy‐confirmed MASH with F2 to F3	50 mg PO QD versus placebo for 52 weeks	50 mg *n* = 112; placebo *n* = 56 (total *n* = 168)	NAS reduction ≥2 without worsening fibrosis or MASH resolution with NAS reduction ≥2 and without worsening fibrosis	NAS reduction ≥2 without worsening fibrosis: 38% versus 16%; MASH resolution with NAS reduction ≥2 and without worsening fibrosis: 26% versus 11%.	PMID: 39396529^78^
Pan‐PPAR agonist	Lanifibranor	2b	Biopsy‐confirmed MASH without F4	1200 mg PO QD; 800 mg PO QD versus placebo for 24 weeks	1200 mg *n* = 83; 800 mg *n* = 83; placebo *n* = 81 (total *n* = 247)	SAF‐A score reduction ≥2 without worsening fibrosis	1200 mg met the primary end point: 55% versus 33%; 800 mg did not meet: 48% versus 33%	PMID: 34670042^79^

Abbreviations: FASN, fatty acid synthase; FGF21, fibroblast growth factor 21; GIP, glucose‐dependent insulinotropic polypeptide; GLP‐1RA, glucagon‐like peptide‐1 receptor agonist; MASH, metabolic dysfunction‐associated steatohepatitis; NAS, NAFLD activity score; PO, per os; PPAR, peroxisome proliferator activated receptor; QW, once a week; Q2W, once every 2 weeks; SAF‐A, the activity part of the Steatosis, Activity, Fibrosis; SC, subcutaneous; SGLT2, sodium‐glucose cotransporter 2; T2D, type 2 diabetes; THR‐β, thyroid hormone receptor beta.

While the choice of these new and emerging pharmacological treatment options for different patient phenotypes needs to be confirmed in clinical trials and real‐world studies, their mode of action may already inform some preferences: Pharmacological agents that primarily reduce hepatic lipid accumulation may be particularly appropriate for hepatic‐predominant MASLD phenotypes, such as liver‐selective THRβ agonists (e.g., resmetirom). In contrast, therapies that predominantly improve systemic metabolic dysfunction may be more suitable for “systemic MASLD,” exemplified by GLP‐1 receptor agonists such as semaglutide. In addition, emerging data indicate that GLP‐1 RAs and FGF21 analogues can reduce alcohol consumption, implying their potential role in managing MetALD.^79^, ^80^

Besides MASH‐directed treatments, the choice of medications for cardiometabolic risk factors and co‐morbidities can have a positive impact on liver health, and those drugs with positive liver effects should be prioritised for their respective indications: Statins are generally safe in MASLD and should be used according to standard thresholds (e.g., ≥10% 10‐year risk, or ≥7.5% where applicable), aiming for intensive LDL‐C lowering in secondary prevention.^58^ Emerging observational data suggest that lipophilic statins (e.g., atorvastatin) may be associated with a lower long‐term risk of HCC and hepatic decompensation in chronic liver disease^81^; however, statins should still be prescribed primarily for cardiovascular risk reduction, in accordance with current guidelines. Healthcare providers should treat hyperglycaemia to standard targets using agents with cardiovascular and renal benefits, but prioritising those beneficial for liver health and weight control (metformin, GLP‐1 receptor agonists, SGLT2 inhibitors, or pioglitazone when appropriate).^82^ Sustained weight loss remains foundational but relapse is common; pharmacotherapy with incretin‐based agents can achieve ~15%–20% weight reduction and favourably impact multiple risk domains.^58^ Last but not least, holistic care should deploy reno‐protective strategies, including blood pressure control using renin‐angiotensin system (RAS) blockade where indicated and SGLT2 inhibitors in eligible patients.^61^

### 4.3 Metabolic and bariatric surgery

In patients with advanced MASH and severe obesity, bariatric surgery should be evaluated according to disease stage and risk profile and is indicated in patients with BMI ≥40 kg/m^2^ (or ≥35 kg/m^2^ with metabolic complications) (Figure 5). Bariatric surgery induces strong and sustained weight loss and can achieve MASH resolution in up to 80% and fibrosis regression in about 40% after 5 years, and it displayed stronger therapeutic effects than lifestyle interventions, without deaths or life‐threatening complications.^83^ Also, bariatric surgery has demonstrated a risk reduction for MALO in compensated cirrhosis related to MASH in a retrospective analysis, implicating it as a potential option for cirrhosis in well‐selected cases.^84^ Besides, endoscopic bariatric interventions (e.g., endoscopic sleeve gastroplasty, duodenal mucosal resurfacing) are emerging for patients unfit for surgery, but their effects on MASH are not well studied.^85^, ^86^

## 5 PATIENT CARE INTEGRATION AND CLINICAL DECISION PATHWAYS IN MASLD

### 5.1 Integrated models of multiprofessional and individualised care

MASLD management demands a holistic, pathway‐based approach, where responsibilities are shared rather than fragmented.^4^, ^87^ Primary care usually plays the first‐line and pivotal role in patient screening, risk stratification and early intervention. The community‐based LOCATE model substantially shortened the time to VCTE assessment by nearly 1 year and suggested a trend towards faster identification of high‐risk MASLD cases.^88^ Furthermore, multiprofessional intensive lifestyle interventions, including a very low‐energy diet, behavioural therapy, dietary counselling, and structured physical exercise, in a real‐world cohort of morbidly obese patients led to substantial weight loss in most participants and was accompanied by marked reductions in ALT and non‐invasive markers of liver steatosis, MASH risk, and fibrosis.^89^ In another multiprofessional tertiary MASLD clinic involving hepatologists, liver nurses and clinical dietitians, most patients maintained or lost weight over 12 months and showed significant improvements in liver enzymes and liver stiffness despite no major change in overall body weight or metabolic risk factors.^90^ Also, a UK MASLD clinic jointly led by hepatology and diabetology with a multiprofessional weight management program to improve diet, lifestyle and exercise, achieved meaningful improvements in ALT, body weight, HbA1c, cholesterol, liver stiffness, and cardiovascular risk over a median 13‐month follow‐up, particularly among patients with T2D.^91^ Although the general evidence level for such multiprofessional interventions remains low, these findings underline the value of multiprofessional teams in improving MASH and fibrosis risk in high‐risk populations.

**FIGURE 6:**
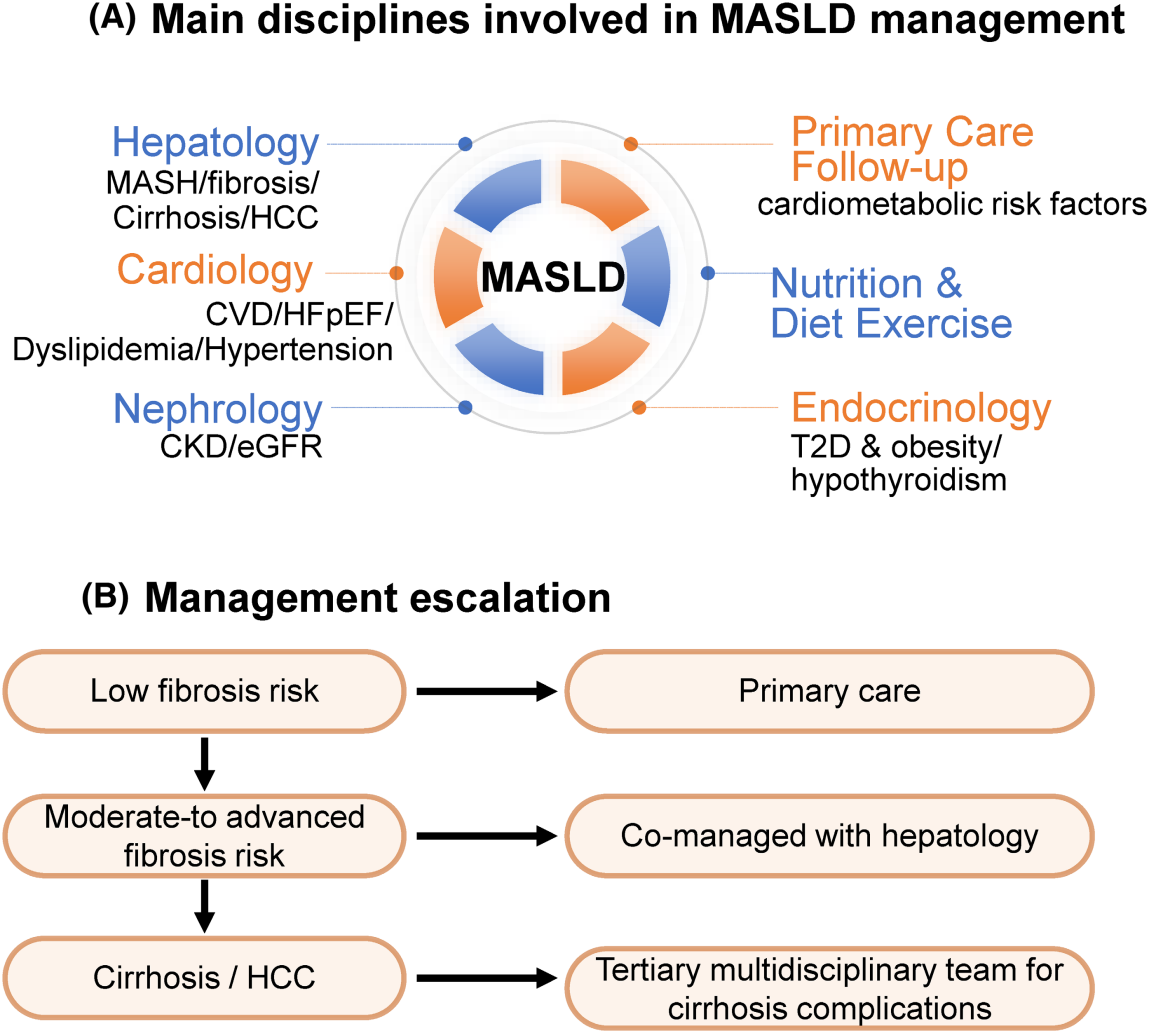
Holistic management of MASLD. (A) Main disciplines involved in MASLD management: This panel illustrates the multisystem nature of MASLD and the corresponding clinical domains that should be integrated into care. Beyond hepatology, effective management requires collaboration with primary care, endocrinology (T2D, obesity, thyroid disorders), cardiology (CVD, HFpEF, dyslipidaemia, hypertension), nephrology (CKD and eGFR monitoring), nutrition and dietetics, and metabolic/bariatric surgery. Each discipline contributes to addressing hepatic, metabolic, cardiovascular, and renal risks, forming the foundation of holistic care. (B) Management escalation: This panel outlines how care intensity increases according to fibrosis severity and clinical complexity. Low fibrosis risk requires long‐term follow‐up within primary care with periodic fibrosis reassessment. Advanced fibrosis risk requires shared management between hepatology and other specialties, including initiation of approved pharmacotherapies and structured metabolic care. Cirrhosis or HCC requires management transitions to a tertiary multidisciplinary team, including hepatology, oncology, interventional radiology, and transplant services for surveillance and management of cirrhosis complications. CKD, chronic kidney disease; CVD, cardiovascular disease; eGFR, estimated glomerular filtration rate; HCC, hepatocellular carcinoma; HFpEF, heart failure with preserved ejection fraction; MASLD, metabolic dysfunction‐associated steatotic liver disease; T2D, type 2 diabetes.

Primary care serves as the entry point for case finding and long‐term follow‐up and is well equipped to diagnose and treat common cardiometabolic conditions such as T2D, dyslipidaemia, and hypertension, and can refer to medical professionals such as behavioural therapists, nutritionists and physiotherapy as needed. However, several aspects of MASLD extend beyond routine primary care and require specialist involvement. Hepatology is essential for diagnostic clarification when liver tests are abnormal, for advanced non‐invasive fibrosis assessment and interpretation of indeterminate results, and for managing patients with suspected MASH, moderate‐to‐advanced fibrosis, cirrhosis, or complications such as portal hypertension or HCC.^4^ Alongside primary care physicians, other specialists such as endocrinologists and nephrologists are well equipped to manage the majority of cardiometabolic risk factors in patients with MASH. This includes initiation and monitoring of incretin‐based therapies (e.g., GLP‐1 receptor agonists), optimisation of obesity and diabetes care, lipid‐lowering and antihypertensive treatment, and integrated cardiometabolic risk reduction. Nephrology input is specially required for patients with advanced chronic kidney disease, albuminuria, or declining renal function, given the close and bidirectional links between CKD and MASLD progression.^61^ Finally, metabolic (bariatric) surgeons evaluate individuals with severe obesity or advanced MASH who may benefit from surgical or endoscopic metabolic interventions.^85^ Together, referral to hepatology, cardiology, endocrinology, or nephrology is recommended primarily in the setting of clinical complexity, including diagnostic uncertainty, suspected or confirmed advanced liver fibrosis or cirrhosis, refractory cardiometabolic risk factors despite guideline‐directed therapy, progressive chronic kidney disease, or when advanced therapeutic decisions are being considered (e.g., bariatric or metabolic procedures, advanced cirrhosis management, or complex cardiovascular disease) (Figure 6A).

### 5.2 Escalation and long‐term follow‐up

The escalation pathway refers to the level and intensity of clinical management, which should increase according to fibrosis stage and the emergence of complications rather than by specialty alone. We propose a three‐stage escalation pathway in MASLD patient management according to the risk of fibrosis, moderate‐to‐advanced fibrosis and cirrhosis (Figure 6B):

1. Low‐risk individuals, including those with no or mild fibrosis on non‐invasive testing, can remain in primary care with reinforcement of lifestyle measures and periodic reassessment of fibrosis, typically every 1–3 years.^4^ This approach minimises unnecessary specialist involvement while ensuring long‐term surveillance for disease progression.
2. Patients at moderate‐to‐advanced fibrosis or showing evidence of fibrosis progression benefit from shared management between primary care and hepatology. Hepatology input helps refine diagnosis, confirm the presence of MASH or advanced fibrosis, and evaluate potential pharmacotherapy when indicated. Cardiometabolic optimisation continues in primary care, while fibrosis monitoring follows hepatology guidance.
3. Cirrhosis does not universally require transition to tertiary care. Many patients with compensated MASH cirrhosis remain clinically stable for years and can be safely co‐managed by hepatology and primary care, provided they undergo routine surveillance for varices, portal hypertension, and hepatocellular carcinoma (HCC).^4^ Escalation to specialist liver centres is needed for patients with decompensated cirrhosis, those developing HCC, or individuals with complex complications requiring transplant evaluation or advanced oncologic/interventional management. This targeted approach ensures that tertiary resources are focused on patients with the highest need while maintaining continuity of care for stable individuals in outpatient settings (Figure 6B). In case of decompensated cirrhosis, HCC or liver failure due to MASLD/MASH, liver transplantation (LT) is recommended, and the primary aetiology of chronic liver disease requiring LT is shifting from viral hepatitis to MASH.^92^ MASLD‐related HCC tends to arise in older, obese/T2D individuals and may show distinct molecular profiles and therapeutic responses compared with HBV/HCV‐related HCC.^93^ Of note, a recent study implicated that patients with diagnosis of MASLD had the lowest survival rate after LT compared with other liver diseases.^92^

## 6 CHALLENGES AND FUTURE DIRECTIONS

### 6.1 Translating disease heterogeneity into clinical practice

MASLD represents a heterogeneous spectrum driven by diverse genetic, metabolic, and environmental factors. Current management approaches remain largely uniform, failing to account for individual variability. Future efforts should focus on precision risk stratification using genomic and metabolic data to predict disease progression and guide personalised interventions, particularly in a feasible and cost‐efficient way.

### 6.2 Establishing and sustaining patient care models

Integrating hepatologists, endocrinologists, cardiologists, nutritionists, and behavioural experts into one team remains a major challenge. Although multidisciplinary care may be beneficial in selected patients, hepatologists, endocrinologists, and cardiologists share substantial overlap in managing cardiometabolic risk, and clinical guidance should therefore focus on defining when referral to a specific specialist is necessary rather than advocating broad multidisciplinary involvement. Besides, healthcare systems in many countries lack both structural support and funding for such programs. Reimbursement models typically reward episodic specialist care rather than long‐term, team‐based management. Sustainable value‐based payment systems and digital coordination platforms could help bridge these gaps and improve accessibility, especially in resource‐limited settings.

### 6.3 Integrating pharmacological therapy with lifestyle modification

Lifestyle change remains the cornerstone of MASLD management, but long‐term adherence is poor. As novel pharmacotherapies targeting steatosis, inflammation, and fibrosis become available, pragmatic strategies are needed to combine drug therapy with sustainable lifestyle interventions. Multimodal programs supported by digital health tools (e.g., smartphone applications and wearable sensors) may enhance adherence and clinical outcomes.^94^, ^95^

### 6.4 Transforming public and professional perception

MASLD remains underrecognised by both clinicians and the public, particularly in low‐ and middle‐income countries. It is often perceived as benign or self‐inflicted, leading to stigma, delayed diagnosis, and care. Educational initiatives and public health campaigns should reframe MASLD as a serious metabolic disorder and integrate it into non‐communicable disease prevention frameworks.

## 7 CONCLUSION

MASLD is a heterogeneous multisystem metabolic disorder where a stepwise, individualised, integrated and coordinated care model is pivotal in optimising patient outcomes, with management led in primary care and escalation to targeted specialist involvement only when clinically indicated by disease severity, diagnostic complexity, or therapeutic need.

## AUTHOR CONTRIBUTIONS

Lanlan Chen drafted the manuscript and drew the figures. Paul Horn substantially revised the manuscript and figures. Frank Tacke designed the review, substantially revised it and supervised the entire research.

## CONFLICT OF INTEREST STATEMENT

Frank Tacke's lab has received research grants (funding to the institution) from AstraZeneca, MSD, Gilead, Agomab. Frank Tacke has received honoraria for consulting or lectures from Gilead, Abbvie, Falk, AstraZeneca, Boehringer, Madrigal, MSD, GSK, Ipsen, Pfizer, Novartis, Novo Nordisk, Sanofi. Paul Horn reports research grants from MSD and Novo Nordisk (to the institution), honoraria for lectures from Falk and Orphalan, and travel support from Falk and Ipsen. Lanlan Chen reports no potential conflict of interest.

## Data Availability

All data are collected from the published clinical trials.
